# A Bacterially Expressed SARS-CoV-2 Receptor Binding Domain Fused With Cross-Reacting Material 197 A-Domain Elicits High Level of Neutralizing Antibodies in Mice

**DOI:** 10.3389/fmicb.2022.854630

**Published:** 2022-04-26

**Authors:** Liqin Liu, Tingting Chen, Lizhi Zhou, Jie Sun, Yuqian Li, Meifeng Nie, Hualong Xiong, Yuhe Zhu, Wenhui Xue, Yangtao Wu, Tingting Li, Tianying Zhang, Zhibo Kong, Hai Yu, Jun Zhang, Ying Gu, Qingbing Zheng, Qinjian Zhao, Ningshao Xia, Shaowei Li

**Affiliations:** ^1^State Key Laboratory of Molecular Vaccinology and Molecular Diagnostics, School of Life Sciences, School of Public Health, Xiamen University, Xiamen, China; ^2^National Institute of Diagnostics and Vaccine Development in Infectious Diseases, Xiamen University, Xiamen, China

**Keywords:** SARS-CoV-2, receptor binding domain, CRM197 A domain, *Escherichia coli* expression system, adjuvant, vaccine

## Abstract

The Coronavirus disease 2019 (COVID-19) pandemic presents an unprecedented public health crisis worldwide. Although several vaccines are available, the global supply of vaccines, particularly within developing countries, is inadequate, and this necessitates a need for the development of less expensive, accessible vaccine options. To this end, here, we used the *Escherichia coli* expression system to produce a recombinant fusion protein comprising the receptor binding domain (RBD) of severe acute respiratory syndrome coronavirus 2 (SARS-CoV-2; residues 319–541) and the fragment A domain of Cross-Reacting Material 197 (CRM197); hereafter, CRMA-RBD. We show that this CRMA-RBD fusion protein has excellent physicochemical properties and strong reactivity with COVID-19 convalescent sera and representative neutralizing antibodies (nAbs). Furthermore, compared with the use of a traditional aluminum adjuvant, we find that combining the CRMA-RBD protein with a nitrogen bisphosphonate-modified zinc-aluminum hybrid adjuvant (FH-002C-Ac) leads to stronger humoral immune responses in mice, with 4-log neutralizing antibody titers. Overall, our study highlights the value of this *E. coli*-expressed fusion protein as an alternative vaccine candidate strategy against COVID-19.

## Introduction

Coronavirus disease 2019 (COVID-19), caused by the severe acute respiratory syndrome coronavirus 2 (SARS-CoV-2), has resulted in more than 271.9 million infections and 5.3 million deaths worldwide (World Health Organization, 2022). In addition, its rapid rate of mutation has resulted in various highly contagious viral strains, which has led to a steady global rise in rates of infection and thus, a unique challenge to human health and public safety. At the time of writing, several vaccines are available; yet, the supply of vaccines does not meet the demand, particularly in developing countries. An inexpensive accessible vaccine is thus urgently needed.

SARS-CoV-2 belongs to the β-coronavirus genus, along with other highly pathogenic—but far less contagious—virus strains, including SARS-CoV, responsible for the “SARS” epidemic in Asia in 2002–2003, and MERS-CoV, responsible for the outbreak in the Middle East about a decade later (Zhu et al., 2020). Like SARS-CoV, SARS-CoV-2 uses the receptor binding domain (RBD) of the spike protein (S) to bind to the receptor angiotensin converting enzyme 2 (ACE2) on host cells for virus entry and subsequent pathogenesis (Hoffmann et al., 2020; Yan et al., 2020; Zhou et al., 2020). The SARS-CoV-2 RBD is immunodominant, containing multiple antigenic sites and accounting for 90% of serum neutralizing activity (Piccoli et al., 2020). Indeed, it triggers the production of potent functional antibodies that play a critical role in immunoprophylaxis, thus making the SARS-CoV-2 spike protein an ideal target for the development of therapeutics against COVID-19 (Du et al., 2020; Liu et al., 2020; Shi et al., 2020). Currently, many different vaccine strategies are employed in the fight against SARS-CoV-2, include recombinant vectors, DNA, mRNA in lipid nanoparticles, inactivated viruses, live attenuated viruses and protein subunits (Krammer, 2020; Draft Landscape of COVID-19 Candidate Vaccines, 2022). Recombinant protein subunit vaccines are particularly advantageous, with proven safety and compatibility and the option of using multiple booster vaccinations where necessary (Jeyanathan et al., 2020). Furthermore, in some cases, the proteins making up the subunits can be prepared using recombinant molecular techniques.

Despite a comprehensive effort to develop RBD-based vaccines, the use of the RBD subunit as a vaccine candidate remains hindered by its limited immunogenicity (Wang et al., 2020). Improving the immunogenicity of RBD requires the use of an appropriate adjuvant or optimization of the protein sequence, fragment length or immune program (Li et al., 2020). CRM197 (Cross-Reacting Material 197), a non-toxic mutant of diphtheria toxin (DT), is widely used as a carrier protein in polysaccharide vaccines (Giannini et al., 1984; Malito et al., 2012). Studies have shown that CRM197 increases the production of Th1- and Th2-secreting T cells during the immune response, and induces B cell proliferation and the secretion of antigen-specific antibodies, thereby enhancing the immunogenicity of that to which it is conjugated (Dagan et al., 2010). Of particular note, the well-studied C-terminal catalytic domain A (aa 1-191) of CRM197 (CRMA) alone has been shown to significantly enhance the immunogenicity of the Hepatitis E virus pORF2-E2 protein and human papillomavirus (HPV) major capsid protein L2 peptide (Wang et al., 2015, 2019). Thus, we posited that the C-terminal catalytic domain A of CRM197 could serve as an intra-molecular adjuvant for RBD to improve its immunogenicity.

The *Escherichia coli* (*E. coli*) expression system has been widely used in the production of numerous recombinant protein drugs due to its rapid growth rate and well-defined genetic profile, as well as the lower costs associated with its culture (Huang et al., 2012). Furthermore, the *E. coli* expression system allows for the rapid expression and cost-effective scale-up of recombinant proteins during manufacturing. Recent years has witnessed the appropriate use of the *E. coli* expression system for the production and subsequent approval of genetically engineered vaccines (e.g., hepatitis E vaccine, human papillomavirus vaccine, meningococcal vaccine; Birkett et al., 2002; Proffitt, 2012; Skibinski et al., 2013; Shirley and Taha, 2018; Masignani et al., 2019; Qiao et al., 2020), including the VLP-based vaccine fully derived from *E. coli*, Hecolin, which was approved for use in humans in 2012 as the first vaccine against Hepatitis E (Proffitt, 2012). Such VLP-based vaccines derived from *E. coli* are highly cost-effective as compared with vaccines derived from insect cells or yeast (Huang et al., 2017). Indeed, insect cells and other expression systems are slower, demand higher costs, and have inherent issues with scalability for commercial purposes. Although not without its limitations, the *E. coli* expression system shows promise for the development of other vaccine types.

Here, we sought to evaluate a candidate vaccine produced using the *E. coli* expression system based on the RBD domain of SARS-CoV-2 fused with CRMA and purified by chromatography. We investigated the physiochemical properties and immunogenicity of this vaccine candidate in a mouse model, and highlight the immune response of CRMA-RBD combined with different adjuvants. Collectively, our findings provide the groundwork for the design of a safe and effective SARS-CoV-2 vaccine based on the RBD using *E. coli*.

## Materials and Methods

### Sera Sample

Six COVID-19 convalescent human sera samples collected from COVID-19 patients after they had recovered from the disease in the First Affiliated Hospital of Xiamen University. Two control sera collected from non-infected persons. Written informed consent was obtained from all volunteers.

### Monoclonal Antibodies

Five monoclonal antibodies (mAbs) were prepared as previously reported (Li et al., 2021; Zhang et al., 2021a): 36H6, 6D6, 7D6, REGN10933, and S309. The 36H6, 7D6, and 6D6 mAbs were screened by immunizing mice with SARS-CoV-2 RBD protein or spike protein alone and in combination with the SARS-CoV spike protein and the MERS-CoV RBD protein, as previously described (Li et al., 2021; Zhang et al., 2021a).

The REGN10933 and S309 mAbs were expressed from Chinese hamster ovary (CHO) cells transiently transfected with plasmids expressing the heavy and light chains, as previously described (Li et al., 2021).

### Gene Cloning, Protein Expression, and Purification

The SARS-CoV-2 subunit antigen, designated CRMA-RBD, comprises residues Arg319–Phe541 of the RBD protein (NCBI accession No. YP009724390) and the catalytic domain A of CRM197 (GeneBank: AMV91693.1), joined by a flexible linker (GGGGSGGGGSGGGGS). To construct plasmids for the expression of RBD, the RBD genes were amplified from synthesized SARS-CoV-2 S gene (Sangon Biotech, Shanghai, China) with primers RBD-F and RBD-R (primer sequence refer to Supplementary Table S1), ligated into products that were amplified with primers pTO-T7-VF and pTO-T7-VR using a nonfusion pTO-T7 expression vector (Wang et al., 2019) as a PCR template, as per the “Gibson Assembly” method (Wang et al., 2019). Similarly, to construct plasmids for the expression of CRMA-RBD, the RBD genes were amplified with primers CRMA-L-RBD-F and CRMA-L-RBD-R, ligated into products that were amplified with primers pTO-T7-VF and pTO-T7-CRMA-L-R using a pTO-T7-CRMA expression vector (Wang et al., 2019) as a PCR template. Then, two recombinant plasmids, pTO-T7-CRMA-RBD and pTO-T7-RBD were separately transferred into the ER2566 *E. coli* strain (Invitrogen) for expression. The 0.5 L *E. coli* ER2566 was grown in LB with 100 μg/ml kanamycin to OD_600_ of 0.6 and induced with 0.1 mM IPTG for 10 h at 24°C.

The expressed CRMA-RBD and RBD proteins were found in form of inclusion body. First, inclusion bodies were separated and purified from cellular debris. The pellets harvested by centrifugation were washed twice with buffer I (50 mM Tris-HCl, 2% Triton X-100, and pH 8.0) and buffer II (50 mM Tris-HCl, pH 8.0), respectively, and then dissolved in buffer III (50 mM Tris-HCl, 8 M urea, and pH 8.0). Next, the CRMA-RBD protein was purified by a three-step chromatography purification process using Q-sepharose (GE Healthcare), SP-sepharose (GE Healthcare), and Phenyl-sepharose (GE Healthcare). The RBD protein was purified by one-step Q-sepharose (GE Healthcare). The chromatography produced an CRMA-RBD protein of ~90% purity (yield, 0.8 mg/L cell culture), and an RBD protein of ∼90% purity (yield, 1.0 mg/L cell culture). Final, two proteins were renatured by dialysis against phosphate buffer (PB; 20 mM pH 7.4, 0.3 M NaCl) at 4°C.

### SDS-PAGE and Western Blotting

Purified proteins were mixed with an appropriate amount of 5× loading buffer, heated at 100°C for 10 min, and separated using 4–12% polyacrylamide tris-glycine gels for 45 min at 180 V. The gels were stained with Coomassie brilliant blue for 30 min at room temperature, destained with KCl_2_ buffer to a clear background, and imaged using the ChemiDoc system (BioRad). For immunoblotting, the gels were then transferred onto nitrocellulose membranes (Whatman, Dassel, Germany). The membranes were blocked and then incubated with an RBD-specific mouse mAb antibody (36H6, 1 μg/ml) at room temperature for 1 h, and washed with PBS containing 0.2% Tween-20 (PBST). Subsequently, the membranes were incubated with horseradish peroxidase (HRP)-conjugated goat anti-human IgG (GAM-HRP) secondary antibody (Abcam, Cambridge, United Kingdom, 1:5,000 dilution), washed again, and then developed using SuperSignal ELISA Pico Chemiluminescent Substrate Kit (Thermo Fisher Scientific).

### High-Performance Size-Exclusion Chromatography

The homogeneity of the CRMA-RBD and RBD proteins was determined by high-performance size-exclusion chromatography (HPSEC; Agilent Technologies 1,200 series; Santa Clara, CA) using a TSK Gel G5000PWXL7.8 × 300 mm column (TOSOH, Tokyo, Japan) equilibrated in 20 mM PB, pH 7.4, containing 0.3 M NaCl. The system flow rate was maintained at 0.5 ml/min and proteins in the eluents were detected at 280 nm.

### Analytical Ultracentrifuge

The sedimentation coefficients for CRMA-RBD and RBD were determined by sedimentation velocity experiments using a Beckman XL-A analytical ultracentrifuge (AUC; Beckman Coulter, Fullerton, CA). Samples were diluted to 0.5 mg/ml in 20 mM PB7.4, 0.3 M NaCl. The AN-60 Ti rotor (Beckman Coulter; Fullerton, CA) speed was set to 40,000 ~ 45,000 rpm for the highest resolution, and the experiment was carried out at 20°C. Data were collected using SEDFIT computer software, kindly provided by Dr. P. C. Shuck (NIH, Bethesda, MA, United States). Multiple curves were fit to calculate the sedimentation coefficient (S) using the continuous sedimentation coefficient distribution model [c (S)], with the c (S) value used to estimate protein molar mass.

### Enzyme-Linked Immunosorbent Assay

ELISA was performed to determine the antigenicity of the CRMA-RBD and RBD proteins. The wells of 96-well microplates were coated with CRMA-RBD, RBD, RBD (Bac) and S-2P (Bac) proteins at 100 ng per well. RBD (Bac) and S-2P (Bac) proteins were both prepared using the insect baculovirus (Bac) expression vector system. After incubation at 37°C for 2 h, the wells were blocked. For RBD-specific neutralizing antibodies (nAbs) detections, 2 μg/ml or 10 μg/ml of nAbs REGN10933, 6D6, 7D6, or S309 were added and serially diluted. For human sera detections, serial dilutions of six convalescent sera and sera from two healthy persons at a starting concentration of 1:50 were added and incubated at 37°C for 0.5 h. The wells were washed and then incubated with HRP-conjugated goat anti-mouse IgG antibody diluted 1:5,000 in assay diluent for measurements of 6D6 and 7D6, and HRP-conjugated goat anti-human IgG antibody for measurements of REGN10933, S309, and human sera. Wells were washed again and the reaction catalyzed using o-phenylenediamine (OPD) substrate at 37°C for 10 min. The optical density (OD_450/630_ nm) was measured on a microplate reader (TECAN, Männedorf, Switzerland).

ELISA was also performed to determine sera antibody binding titers. Mouse serum samples were serially diluted 3-fold from a starting concentration of 1:50 or 1:100 and pipetted into the wells of antigen-coated plates. The plates were then incubated for 30 min at 37°C. The plates were washed, and then incubated for 30 min at 37°C with 100 μl/well of a 1:5,000 diluted goat anti-mouse IgG-HRP antibody. The chromogenic reaction and optical density (OD_450/630_ nm) detection methods are the same as the procedures described above. The cut-off value was calculated as the mean of the control OD_450_ nm value plus three times the SD (STDEV.P) of the pre-immunization serum samples (*n* = 40). The binding titer was defined as the first dilution with a value larger than the cut-off value.

### Surface Plasmon Resonance

Human ACE2–hFc was captured to ~100 response units (RUs) on Sensor Chip Protein A. For kinetic analysis, CRMA-RBD and RBD proteins were run across the chip in a 2-fold dilution series (12.5, 25, 50, 100, 200, 400, 800, and 1,600 nM), with another channel set as the control. Each sample that had bound to the antigen surface was dissociated by PBS-P+ running buffer (Cytiva) for 120 s at a flow rate of 30 μl/min. Regeneration of the sensor chips was performed for 60 s using regeneration buffer (glycine pH1.5). The resulting data were fitted to a 1:1 binding model using Biacore Evaluation Software (GE Healthcare). Surface plasmon resonance (SPR)-based measurements were performed using a Biacore 8K (GE Healthcare).

### Vaccine Formulation

Each antigen [the CRMA-RBD, RBD, or RBD (Bac) proteins] was absorbed to an equal volume of Alum adjuvant (Al-001-840, the Al content in the formulation is 0.84 mg/ml) or a nitrogen bisphosphonate-modified zinc-aluminum hybrid adjuvant (FH-002C-Ac; Wu et al., 2021) to achieve the desired concentration [6.67 μg/ml (1 μg per dose) or 33.33 μg/ml (5 μg per dose)] in final formulation.

For the Freund’s adjuvant-based formulation, equal volume of antigens and complete Freund’s adjuvant (Sigma-Aldrich; the initial injection) or incomplete Freund’s adjuvant (Sigma-Aldrich; the boost injection) were mixed into a stable emulsion.

### Animals Vaccination

The experimental protocols were approved by the Xiamen University Laboratory Animal Management Ethics Committee. Female, 6-week-old BALB/c mice were purchased from Shanghai SLAC Laboratory Animal Co., Ltd. All animal procedures were approved by the Xiamen University Laboratory Animal Management Ethics Committee. All manipulations were strictly conducted in compliance with animal ethics guidelines and approved protocols.

BALB/c mice were subcutaneously immunized with various antigens (1 μg or 5 μg per dose) combined with Freund’s adjuvant, and intramuscularly immunized with various antigens (1 μg or 5 μg per dose) combined with Alum adjuvant or FH-002C-Ac adjuvant (150 μl per mouse), following an immunization schedule of one priming dose at week 0 plus two boosters at weeks 2 and 4 (*n* = 6 per group). Serum samples were collected at 1-week intervals after immunization *via* retro-orbital bleeding to measure the antigen-specific IgG antibody titers. For cellular immune response analyses, splenocytes cells were collected 7 days after the third immunization for intracellular cytokine staining (ICS) measurements.

To estimate the degree of persistence of the vaccine-induced immune response, BALB/c mice were immunized with various vaccine formulations according to the above three-dose immunization schedule (*n* = 5 per group). Serum samples were collected to measure the antigen-specific IgG antibody titers and neutralizing antibody titers at weeks 0, 1, 2, 3, 4, 5, 6, 7, 8, 12, and 16.

### Pseudovirus-Based Neutralization Assay

Pseudovirus-based neutralization assays were performed as previously reported (Xiong et al., 2020). BHK21-hACE2 cells were pre-seeded in 96-well plates. Serially diluted (3-fold gradient) serum samples were mixed with diluted VSV-SARS-CoV-2-Sdel18 virus and incubated at 37°C for 1 h. The mixture was added to the pre-seeded BHK21-hACE2 cells. After 12 h of incubation, fluorescence images were obtained with Opera Phenix or Operetta CLS equipment (PerkinElmer). For quantitative determination, fluorescence images were analyzed by the Columbus system (PerkinElmer), and the numbers of GFP-positive cells for each well were counted to represent infection performance. The neutralization titer for each sample was expressed as the maximum dilution fold (ID_50_) required to achieve infection inhibition by 50% (50% reduction in GFP-positive cell numbers as compared with controls).

### Flow Cytometry

Splenocytes (2 × 10^6^ per test) harvested from mouse spleen were stimulated with 2 μg/ml pooled peptides of SARS-CoV-2 RBD protein (15-mer peptides with 11–amino acid overlap covering the entire RBD protein; GenScript; refer to Supplementary Table S2) for an 18-h incubation in a CO_2_ incubator. Protein transport inhibitors (BD GolgiPlug; BD Biosciences) were added and incubated for 6 h. Cells were then stained for flow cytometry analysis using the following antibodies from BD Biosciences: FITC Rat Anti-Mouse CD4, PE-Cy7 Rat Anti-Mouse CD8a, and LIVE/DEAD Fixable Aqua Dead Cell Stain Kit. Subsequently, cells were fixed and permeated by using Fixation/Permeabilization Solution Kit, and further stained with APC Rat Anti-Mouse IFN-γ and PE Rat Anti-mouse IL-2. Finally, the samples were measured using a BD LSRFortessa X-20 Flow Cytometer (BD), with the data were analyzed by FlowJo V10.6.0. The gating strategy for detecting T cell subsets is shown in Supplementary Figure S1.

### Statistics

All statistical analyses were performed using GraphPad Prism 9 software (La Jolla, CA), Origin2021, and FlowJo V10. The nonparametric Kruskal–Wallis test was used to analyze the differences among more than two groups and followed by the Dunn’s method correcting for multiple comparisons. *p* values in each group are indicated as follows: ^*^p < 0.05, ^**^p < 0.01, and ^***^p < 0.001.

## Results

### Characterization of the Recombinant CRMA-RBD and RBD Proteins

**Figure 1:**
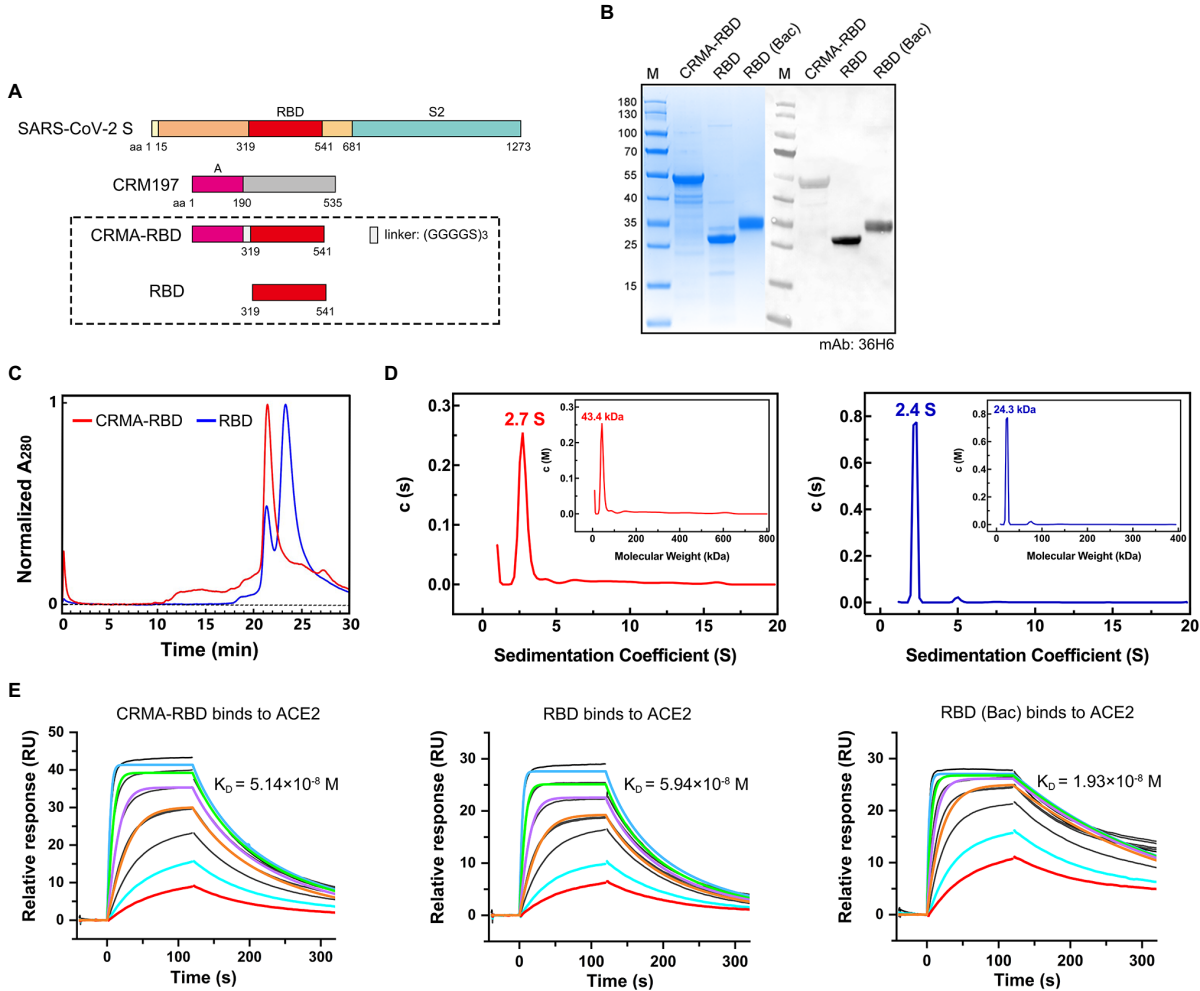
Construct design and characterization of the CRMA-RBD protein. **(A)** Schematic map of the CRMA-RBD construct design. Fragment A of Cross-Reacting Material 197 (CRM197) was fused to the N-terminus of severe acute respiratory syndrome coronavirus 2 (SARS-CoV-2) RBD *via* a flexible linker. S, spike protein; CRM197, Cross-Reacting Material 197; and RBD, receptor binding domain. **(B)** Purified CRMA-RBD and RBD analyzed by SDS-PAGE and western blotting. Bac: Baculovirus expression vector system. RBD (Bac), recombinant RBD protein from Baculovirus insect cell expression. M: Marker. **(C)** Purified CRMA-RBD and RBD proteins were evaluated by high-performance size-exclusion chromatography (HPSEC). **(D)** Purified CRMA-RBD and RBD proteins were analyzed by analytical ultracentrifugation (AUC). CRMA-RBD is shown in red, and RBD is shown in blue. Sedimentation coefficients of CRMA-RBD and RBD were determined by sedimentation velocity (SV) tests; molecular weights of CRMA-RBD and RBD in solution were evaluated by the c (M) method. **(E)** Analysis of CRMA-RBD (left), RBD (middle), and RBD (Bac; right) binding to the ACE2 receptor protein by surface plasmon resonance technology (SPR).

Evidence suggests that the SARS-CoV-2 spike protein triggers the production of potent, functional antibodies that initiate an immunoprophylactic response against COVID-19 (Du et al., 2020; Liu et al., 2020; Shi et al., 2020). We constructed a candidate antigen by fusing the fragment A domain of CRM197 with the RBD (residues 319–541) of the spike protein using a flexible peptide linker, hereafter referred to as “CRMA-RBD” (Figure 1A). Using the *E. coli* expression system, recombinant SARS-CoV-2 CRMA-RBD and RBD proteins were obtained (Figure 1B). Both of the purified proteins showed acceptable purity, with molecular weights (m.w.) of ∼48 kDa, and 25 kDa for CRMA-RBD and RBD, respectively. The CRMA-RBD and RBD proteins reacted well with 36H6 antibodies (Zhang et al., 2021a) in immunoblotting analysis (Figure 1B). Due to the lack of glycosylation modifications in *E. coli*, as expected, the *E. coli*-derived RBD protein has a smaller molecular weight than the RBD protein derived from the baculovirus (Bac) insect cell expression system.

Next, we used HPSEC and AUC to analyze the oligomerization potential of the two recombinant proteins in solution. HPLC analysis showed that CRMA-RBD presented a single major peak with retention time of 21.5 min (Figure 1C). In contrast, RBD showed not only one major peak at about 23.2 min (account for 75.1%), but also a small peak at about 21.5 min (account for 24.9%). In the AUC profiles, for CRMA-RBD, a predominant sedimentation peak was observed at 2.7 S, approximately equivalent to 43.4 kDa (Figure 1D). RBD resolved at 2.4 S, equivalent to 24.3 kDa. Furthermore, RBD had a small peak at 5.0 S, possibly corresponding to the small species resolved in the HPLC profile, which should be minor amount of RBD oligomers. Together, these results showed the CRMA-RBD protein have higher homogeneity in solution than RBD.

To investigate the conformational integrity of the RBD after fusion to the C-terminal CRM197-A fragment, we analyzed the binding affinities of CRMA-RBD and RBD with the ACE2 protein using SPR. As shown in Figure 1E, the equilibrium dissociation constants (K_D_) of CRMA-RBD and RBD proteins for the immobilized hACE2-Fc protein were ~5.14 × 10^−8^ M and 5.94 × 10^−8^ M, respectively, which closely match the binding affinity of RBD (Bac) protein (K_D_ = 1.93 × 10^−8^ M). These results suggest that *E. coli*-derived RBD proteins display a native conformation, and that fusion with CRMA has no effect on the structure of the RBD.

### Antigenicity of CRMA-RBD and RBD Proteins

The antigenicity levels of the *E. coli*-derived CRMA-RBD and RBD proteins were characterized by SARS-CoV-2 nAbs [REGN10933 (Hansen et al., 2020), 6D6, 7D6 (Li et al., 2021), and S309 (Pinto et al., 2020)] and COVID-19 convalescent human sera, with RBD (Bac) or S-2P (Bac) protein serving as controls. Four nAbs were prepared as previously reported (Li et al., 2021). S-2P is a trimer stabilization design of the spike protein encompassing amino acids 15-1,213, and engineered with two proline substitutions at residues 986 and 987 along with the inclusion of an “AGAG” at the furin cleavage site (residues 682–685). The S-2P protein was also expressed using the Baculovirus expression vector system, and is hereafter referred to as S-2P (Bac; Li et al., 2020).

**Figure 2:**
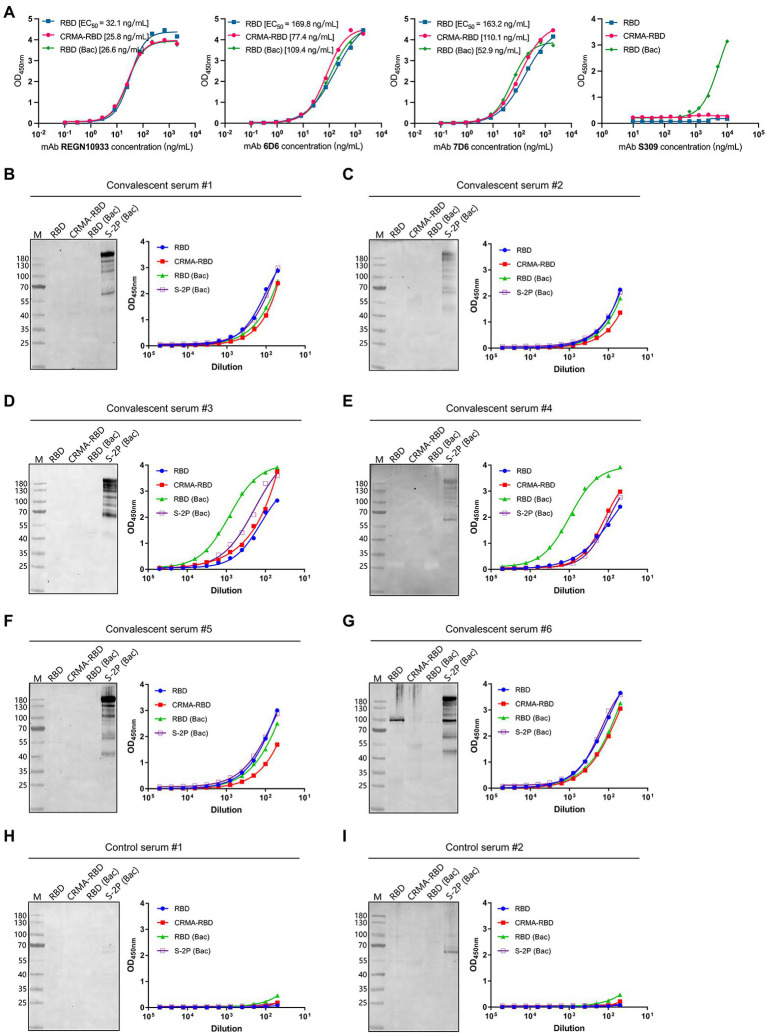
Antigenicity of the CRMA-RBD and RBD proteins against SARS-CoV-2 neutralizing antibodies (nAbs) and Coronavirus disease 2019 (COVID-19) convalescent sera. **(A)** The reactivity of the CRMA-RBD, RBD, and RBD (Bac) proteins with four nAbs (REGN10933, 6D6, 7D6, and S309) determined by ELISA. **(B–G)** The reactivity of the CRMA-RBD, RBD, RBD (Bac) and S-2P (Bac) proteins against the COVID-19 convalescent human sera (#1–#6) as determined using western blotting (left panel) and ELISA (right panel). Convalescent sera were collected from patients with COVID-19 after they had recovered from the disease. **(H,I)** Results of two control sera collected from non-infected persons.

First, the CRMA-RBD and RBD proteins were analyzed by ELISA for their reactivity to four well-characterized nAbs, which recognize separate epitopes within RBD (Li et al., 2021). The CRMA-RBD and RBD proteins reacted well with both REGN10933, 6D6, and 7D6 antibodies, but weakly with S309 compared to RBD derived from insect cell expression (Figure 2A). Crystal structure showed that S309 recognized a highly conserved epitope in the RBD that comprises the N343 glycan, which explains why *E. coli*-derived recombinant RBD protein reacted weakly with S309. Thus, despite the lack of glycosylation in *E. coli* expression system, the *E. coli*-derived CRMA-RBD and RBD proteins maintain the conformational probity of most neutralization epitopes except for some glycan-related one.

To further evaluate the antigenicity of two proteins, we performed ELISA and western blotting using a panel of six COVID-19 convalescent human sera, which collected from COVID-19 patients after they had recovered from the disease in the First Affiliated Hospital of Xiamen University; two control sera collected from non-infected persons were also used. Immunoblotting analysis showed good reactivity of the S-2P (Bac) protein against all six convalescent sera, whereas CRMA-RBD, RBD, and RBD (Bac) proteins showed little to no reactivity. In contrast, in the ELISA-binding assay, we found strong reactivity for the *E. coli*-derived CRMA-RBD and RBD proteins against the six convalescent sera, similar to the RBD (Bac) an S-2P (Bac) proteins (Figures 2B–G, right panel). In addition, no detectable reactions were observed with the control sera (Figures 2H,I).

Collectively, these results suggest that *E. coli*-derived CRMA-RBD and RBD proteins maintain native-like SARS-CoV-2 epitopes. These epitopes in the native virion should be immunogenic in COVID-19 patients and capable of eliciting high antibody titers in the convalescent phase of SARS-CoV-2 infection. Given that most RBD epitope sites are strictly conformation dependent, it is not surprising that mild reducing and denaturing conditions with SDS treatment in the western blotting analysis lead to little or no binding; the NTD S1 and S2 regions [i.e., S-2P (Bac) protein] bear some linear epitopes that would not be as affected by denaturation.

### RBD-Specific Neutralizing Antibody Responses Induced by CRMA-RBD

We next evaluated the immunogenicity of our *E. coli*-derived recombinant vaccine candidates. Mice were immunized with two dosages (1 and 5 μg) of CRMA-RBD, RBD or RBD (Bac; as a control) with Freund’s adjuvant, a standard Alum adjuvant (Al-001-840), or a nitrogen bisphosphonate-modified zinc-aluminum hybrid adjuvant (FH-002C-Ac; Wu et al., 2021). Phosphate-buffered saline (PBS) was used for the negative control group. Each group of mice (*n* = 6 per group) was vaccinated at weeks 0, 2, and 4. To assess the humoral immune response induced by the recombinant protein vaccine candidates, RBD-specific IgG and neutralizing antibody titers were measured by ELISA and a neutralization assay based on vesicular stomatitis virus (VSV) pseudovirus and BHK21 cells expressing ACE2, respectively.

**Figure 3:**
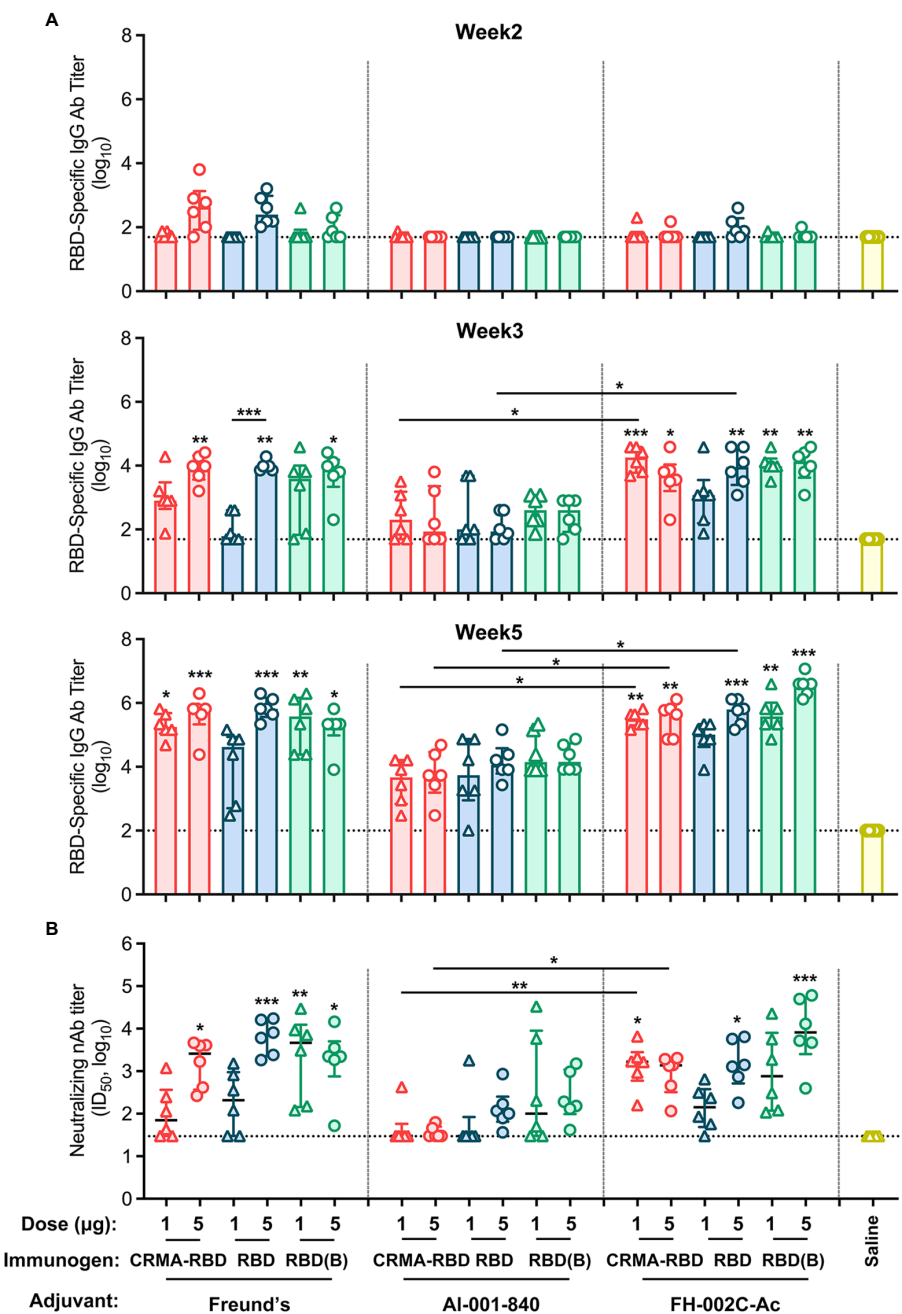
Immunogenicity of CRMA-RBD, RBD, and RBD (Bac) in mice. **(A)** IgG titers induced by CRMA-RBD, RBD, or RBD (Bac) in BALB/c mice (*n* = 6). Mice were immunized with 1 or 5 μg of CRMA-RBD, RBD, or RBD (Bac), delivered with Freund’s adjuvant, Alum adjuvant (Al-001-840) or the FH-002C-Ac adjuvant at weeks 0, 2, and 4. **(B)** Neutralizing antibody titers of the immune sera at week 5, corresponding to the samples showing IgG titers in (**A**, bottom panel). Data were plotted as median ± IQR. The statistical significance was determined by a Kruskal–Wallis test with Dunn’s multiple comparisons test. *p* values: ^*^p < 0.05, ^**^p < 0.01, and ^***^p < 0.001. *p* values are indicated in the plots: *p* values above column corresponding the difference between the group and the saline group; *p* values above line corresponding the difference between two independent groups. RBD (B): RBD (Bac).

For all three proteins, mice immunized with Freund’s and FH-002C-Ac adjuvants showed rapid antibody responses, presenting detectable anti-RBD IgG antibodies at week 2 (Figure 3A, top panel). Compared with the saline-vaccinated group, seroconversion of IgG antibodies occurred in most immunized mice after the second vaccination (Figure 3A, middle panel). For the CRMA-RBD protein, antibody titers were significantly higher when combined with the FH-002C-Ac adjuvant than the Alum adjuvant, with week-5 measurements of ~5.5-log and 3.5-log titers, respectively. Of note, the two dosages of CRMA-RBD formulated with FH-002C-Ac adjuvant induced comparable IgG titers. At low dosages (1 μg), CRMA-RBD elicited higher IgG titers than did the RBD (Figure 3A, bottom panel). In comparison, IgG titers were dose-dependent for the *E. coli*-derived RBD protein with the Freund’s adjuvant but this was not the case for baculovirus-derived RBD protein.

In addition to antibody production intensity, immunization quality critically depends on the neutralization ability of the generated antibodies. After the 3-dose regimen, at week 5, the antibody titers of the CRMA-RBD protein were about 3-log when combined with the FH-002C-Ac adjuvant, similar to that observed with Freund’s adjuvant (Figure 3B). Yet, CRMA-RBD combined with the FH-002C-Ac adjuvant induced more than 2-fold higher neutralizing antibody titers than it did when combined with the Alum adjuvant, implying a potent immunostimulatory effect of the FH-002C-Ac adjuvant. The control group showed only background-level antibody responses, with neither the IgG nor the neutralizing antibody. Together, recombinant CRMA-RBD protein combined with FH-002C-Ac adjuvant could enhance the induction of antibodies with a higher level of specific antibodies at a lower immunization dosage.

### T Cell Response Induced by CRMA-RBD

**Figure 4:**
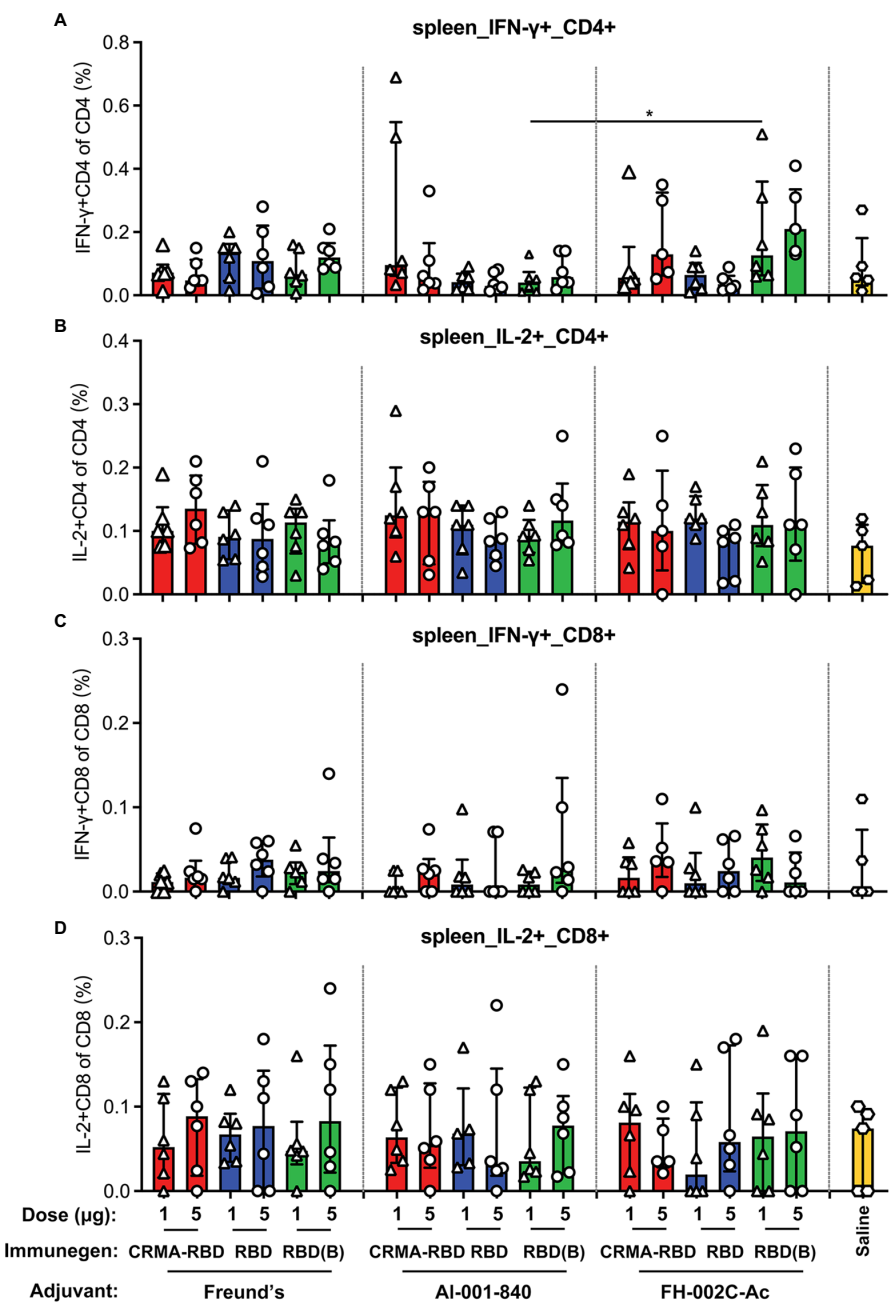
T cell immune responses in BALB/c mice vaccinated with CRMA-RBD vaccine candidate. BALB/c mice (*n* = 6) were immunized with CRMA-RBD, RBD, or RBD (Bac) vaccine candidates at two doses (1 and 5 μg per dose) administered with Freund’s adjuvant, Alum adjuvant, or FH-002C-Ac adjuvant. Splenocytes were collected 7 days after the 3rd immunization and stained for fluorescence activated cell sorting (FACS). The frequencies of IFN-γ^+^CD4^+^ T cells **(A)**, IL-2^+^CD4^+^ T cells **(B)**, IFN-γ^+^CD8^+^ T cells **(C)**, and IL-2^+^CD4^+^ T cells **(D)** are plotted as median ± IQR. The statistical significance was determined by a Kruskal–Wallis test with Dunn’s multiple comparisons test. *p* values: ^*^p < 0.05. RBD (B): RBD (Bac).

Although it is important to establish humoral immunity against SARS-CoV-2 to prevent COVID-19 disease, an early and robust T cell response elicited by the vaccine is also critically important, it may benefit for lasting protection duration as well (Altmann and Boyton, 2020). Thus, we next sought to determine the RBD-specific T cell responses induced by our potential vaccine candidates in immunized mice. Splenocytes collected 7 days after the third immunization were stimulated with SARS-CoV-2 RBD overlapping peptides, and then assessed by flow cytometry analysis for the secretion of IFN-γ^+^ and IL-2^+^ CD4^+^ T cells, as well as IFN-γ^+^ and IL-2^+^ CD8^+^ T cells. CRMA-RBD formulated with either Alum or FH-002C-Ac adjuvant could induce higher frequencies of RBD-specific IFN-γ^+^ CD4^+^ or CD8^+^ T cells, and IL-2^+^ CD8^+^ T cells (Figures 4A,C,D). Similarly, RBD (Bac) generated an increased frequency of IFN-γ–producing CD4^+^ or CD8^+^ T cells (Figures 4A,C). Therefore, CRMA-RBD combined with the Alum adjuvant or the FH-002C-Ac adjuvant can effectively elicit RBD-specific CD4^+^ and CD8^+^ T cell responses.

### Persistence of CRMA-RBD-Induced Immune Response in Mice

**Figure 5:**
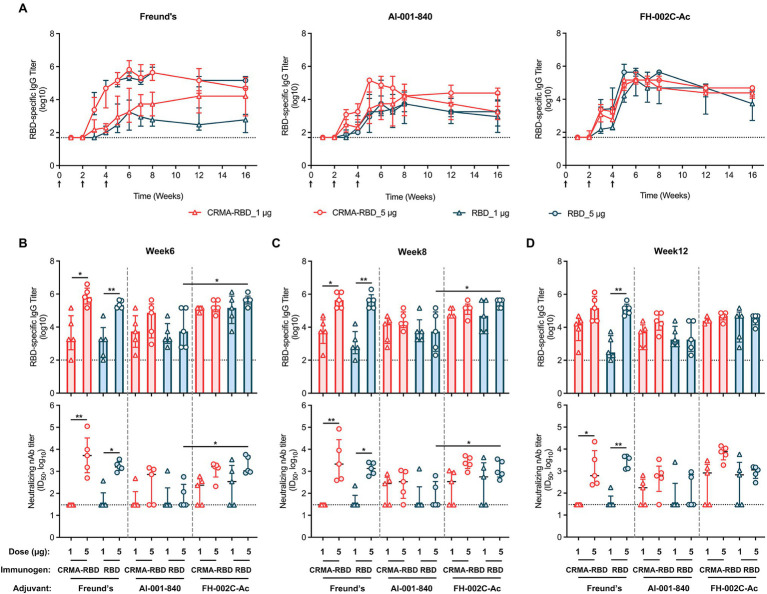
The persistence of recombinant CRMA-RBD-induced humoral immunity in mice. **(A)** IgG titers of anti-RBD in immunized mice were monitored for up to 16 weeks. Mice (*n* = 5) were vaccinated with three doses of CRMA-RBD and RBD antigen formulations. Black arrows indicate the immunization time points. Serum samples were collected at appropriate intervals (weeks 0, 1, 2, 3, 4, 5, 6, 7, 8, 12, and 16) after immunization. **(B–D)** RBD-specific and neutralizing antibody titers measured at weeks 6, 8, and 12 post-vaccination. Data were plotted as median ± IQR. The statistical significance was determined by a Kruskal–Wallis test with Dunn’s multiple comparisons test. *p* values: ^*^p < 0.05 and ^**^p < 0.01.

Finally, we sought to estimate the degree of persistence of the CRMA-RBD-induced immune response in mice by monitoring RBD-specific antibody titers over 16 weeks (Figure 5A). This test was carried out for CRMA-RBD in head-to-head manner with RBD as control, both of which were prepared from *E. coli*. In terms of the results of the first immunization test, the saline group and RBD (Bac) group were omitted.

All of the tested candidate-induced antibody titers rapidly increased after each of the two booster immunizations given at weeks 2 and 4. When formulated with Freund’s adjuvant, the IgG titers for CRMA-RBD were dose-dependent, with up to 5.8-log and 3.3-log for the 5 and 1 μg doses, respectively, at week 6 (Figures 5A,B, left panel); the corresponding neutralizing antibody titers were about 3.7-log and with a consistent trend in the response for the detection limit (Figures 5B–D). Of note, 5 μg CRMA-RBD and RBD induced comparable IgG titers, whereas, at the 1 μg dose, CRMA-RBD elicited a higher IgG titer than RBD over time thereafter (Figure 5A, left panel). In addition, anti-RBD antibodies were also observed at comparable titers in mice immunized with 1 μg CRMA-RBD and 5 μg RBD combined with the Alum adjuvant (Figure 5A, middle panel). In terms of the persistence of antibody response, CRMA-RBD formulated with Freund’s and Alum adjuvants could maintain slightly higher antibody levels over time as compared with RBD.

However, compared with Freund’s and Alum adjuvants, FH-002C-Ac-adjuvanted CRMA-RBD and FH-002C-Ac-adjuvanted RBD stimulated more rapid antibody responses in mice, reaching peak levels (~5.5-log) at week 5, which were maintained through to week 8 (Figure 5A, right panel). Following week 8, the titers slightly decreased to about 4.5-log until week 16. Notably, IgG titers for mice administered with CRMA-RBD–FH-002C-Ac and RBD–FH-002C-Ac were comparable at either dosage. However, mice that received a high dosage (5 μg) of CRMA-RBD–FH-002C-Ac were afforded much higher neutralization titers than those that received the low dose; indeed, some mice in the low-dose group failed to produce neutralizing antibodies, likely due to individual differences. In addition, neutralization antibody titers in the CRMA-RBD high-dose group were maintained at higher levels at week 12 relative to that of RBD protein (Figure 5D, bottom panel). The persistence in the binding and neutralization antibody titer levels over the long-term hints to the considerable longevity of the immunity induced by vaccinating mice with CRMA-RBD combined with the FH-002C-Ac adjuvant.

## Discussion

Vaccines are urgently needed to control the ongoing COVID-19 pandemic. The SARS-CoV-2 spike RBD is an attractive target for vaccine design. In this study, we describe a SARS-CoV-2 subunit vaccine candidate containing the RBD fused to the CRM197 A domain (CRMA-RBD) and expressed in *E. coli*. We show that this fusion CRMA-RBD protein combined with FH-002C-Ac adjuvant can effectively induce neutralizing antibody titers, thus representing a promising pathway for SARS-CoV-2 vaccine development.

Several eukaryotic expression systems, including mammalian cells, baculovirus-insect cells and yeast, have been used to express the recombinant RBD protein (Chen et al., 2020; Dai et al., 2020; Yang et al., 2020; Sinegubova et al., 2021). Yet, the *E. coli* expression system has otherwise advantages for the expression of exogenous proteins, including efficiency, time–cost saving and yield. These differences are particularly relevant in the current urgent circumstances surrounding SARS-CoV-2. However, proteins expressed with *E. coli* lack post-translational modifications, such as glycosylation, and suffering from correct disulfide bond formation sometimes, which are often critical for proper protein folding, stability, and/or activity. Previous studies (Lan et al., 2020) have reported that eight of the nine cysteine residues in the RBD form disulfide bond pairs (Cys336-Cys361, Cys379-Cys432, Cys391-Cys525, and Cys480-Cys488) that help to stabilize the structure. In the present study, we systematically optimized the renaturation conditions of the CRMA-RBD protein, and found it to have a strong interaction with the ACE2 protein (Figure 1E, left panel); this may indicate that the *E. coli*-derived CRMA-RBD maintains overall native conformation. This was further confirmed with ELISA results, as demonstrated good reactivity of CRMA-RBD against several representative nAbs (Figure 2A) and COVID-19 convalescent sera (Figures 2B–G, right panel). Finally, the immunogenicity of CRMA-RBD protein was similar to that of the baculovirus-derived RBD protein, and we found no significant difference in neutralizing antibody titers elicited by these two kinds proteins with FH-002-Ac adjuvant (Figure 3B). Of note, the RBD domain (Arg319-Phe541) contains two N-glycosites (Asn331 and Asn343), which were outside the motifs that are essential for direct interaction with the ACE2 receptor (Zhang et al., 2021b). As expected, the CRMA-RBD and RBD protein produced in *E.coli* reacted weakly with S309 (target N343 glycan) but well with both REGN10933, 6D6, and 7D6 antibodies despite of the lack of glycosylation (Figure 2A), which indicated the *E. coli*-derived proteins maintain overall conformational probity of most neutralization epitopes except for some glycan-related one. Thus, to some extent, our results suggest that glycosylation is not critical for inducing protective immunity using the RBD. Collectively, these features provide alternative options for vaccine design and immune recognition.

Previous studies have shown that CRM197 increases the production of Th1- and Th2-secreting T cells during the immune response and subsequently induces the differentiation and maturation of B cells by heterogeneous cytokines. CRM197 has been used as a carrier protein in several licensed conjugated vaccines, including PREVNAR7, PREVNAR13, and HibTITER (Shinefield, 2010). In these vaccines, the polysaccharide is chemically covalently linked to the CRM197 protein. Our previous study had shown that CRMA itself can serve as an intramolecular adjuvant: the truncated HEV capsid protein (E2) fused with CRMA showed 10-times more immunogenicity than the particulate p239 (Wang et al., 2019). As such, we chose to examine the utility of CRMA conjugation to enhance the immunogenicity of RBD. We showed CRMA-RBD combined with the Alum adjuvant induced a lower antibody titer (Figure 3), but a slightly stronger antigen-specific T cell response compared with RBD (Figures 4A,C), suggesting that the elicited T cell responses to CRMA may also enhance responses to the RBD. In the presence of the FH-002C-Ac adjuvant, both CRMA-RBD and RBD stimulated stronger humoral responses, however, we did not find obviously further enhancement by CRMA. Noteworthy, IFN-γ^+^ CD4^+^ T cells in animals receiving Alum adjuvant seems slightly higher in 1 μg dose group than that in 5 μg dose (Figure 4A). However, these slightly higher response of CRMA-RBD vs. RBD and 1 μg dose vs. 5 μg dose in CRMA-RBD–Alum group show no significantly statistical difference, which might be due to the measurement variation in dividual animal within a group.

The FH-002C-Ac adjuvant offers obvious advantages in terms of inducing a stronger humoral immune response to the CRMA-RBD protein. High titer-specific IgG antibody levels were detected on 1 week after the third immunization. Immunization with CRMA-RBD combined with FH-002C-Ac at low doses rapidly produce anti-RBD and neutralizing antibodies in mice (Figures 3A,B). For FH-002C-Ac adjuvant, the addition of a drug for osteoporosis nitrogen bisphosphonate (risedronate) and zinc ions to the composite alum adjuvant have showed an enhance immunostimulatory effect in our previous study (Wu et al., 2021). Thus, various antigens combined with FH-002C-Ac adjuvant could enhance the induction of antibodies with a higher level of specific antibodies at a lower immunization dosage. We surmise the 1 and 5 μg dose is too high to show the dose dependence, which might require further test in smaller dose. In comparison, the immune stimulation effect of Alum adjuvant is weak, 5 μg CRMA-RBD and RBD induced comparable IgG titers with 1 μg dose. Nonetheless, our data showed that immunization using CRMA-RBD combined with the FH-002C-Ac adjuvant could elicit RBD-specific CD4^+^ and CD8^+^ T cell responses (Figure 4); this would help to establish protective immunity afforded by both arms of the immune response in vaccinated individuals. Overall, the mechanisms of action of many adjuvants, including aluminum salts, FH-002C-Ac adjuvant and CRMA, are still to be fully elucidated.

In summary, we explored a SARS-CoV-2 subunit vaccine candidate based on a recombinant CRMA-RBD expressed in *E. coli*. The CRMA-RBD protein exhibited good physicochemical properties and produce strong immunogenicity response in BALB/c mice when combined with an FH-002C-Ac adjuvant. Our study outlined the expression, purification and characterization of an *E.coli*-derived CRMA-RBD protein, and pave an alternative way to produce a SARS-CoV-2 vaccine with potentially high cost-effective.

## Data Availability Statement

The datasets presented in this study can be found in online repositories. The names of the repository/repositories and accession number(s) can be found at: https://www.ncbi.nlm.nih.gov/, YP009724390 and AMV91693.1.

## Ethics Statement

The animal study was reviewed and approved by Xiamen University Laboratory Animal Management Ethics Committee.

## Author Contributions

SL, QZa, QZe, YG, TL, JZ, and NX conceptualized and designed the study. LL, LZ, QZa, HY, and SL designed the clones and produced the proteins. YL, TL, LZ, WX, YW, and ZK characterized the proteins. LL, TC, MN, LZ, JS, WX, and YG performed the animal experiments. LL, JS, HX, YZ, and QZe performed the ELISA and neutralization assays. LL, TC, TZ, ZK, TL, and JS performed flow cytometry. LL, TC, LZ, QZe, and JZ wrote the paper. SL, QZa, YG, NX, and HY revised the manuscript. All authors contributed to the article and approved the submitted version.

## Funding

This work was supported by the National Natural Science Foundation of China (Grant No. 32070925), the National Key Research and Development Program of China (Grant No. 2021YFC2301404), and the Principal Fund (Grant No. 20720190117).

## Conflict of Interest

The authors declare that the research was conducted in the absence of any commercial or financial relationships that could be construed as a potential conflict of interest.

## Publisher’s Note

All claims expressed in this article are solely those of the authors and do not necessarily represent those of their affiliated organizations, or those of the publisher, the editors and the reviewers. Any product that may be evaluated in this article, or claim that may be made by its manufacturer, is not guaranteed or endorsed by the publisher.
