# Supplementary material for: A Bacterially Expressed SARS-CoV-2 Receptor Binding Domain Fused With Cross-Reacting Material 197 A-Domain Elicits High Level of Neutralizing Antibodies in Mice
Source: Front Microbiol. 2022 Apr 26;13:854630. doi: 10.3389/fmicb.2022.854630 (PMC9087041; doi:10.3389/fmicb.2022.854630)
Supplement: Supplementary file 1 [file Presentation_1.pptx]

## Slide 1
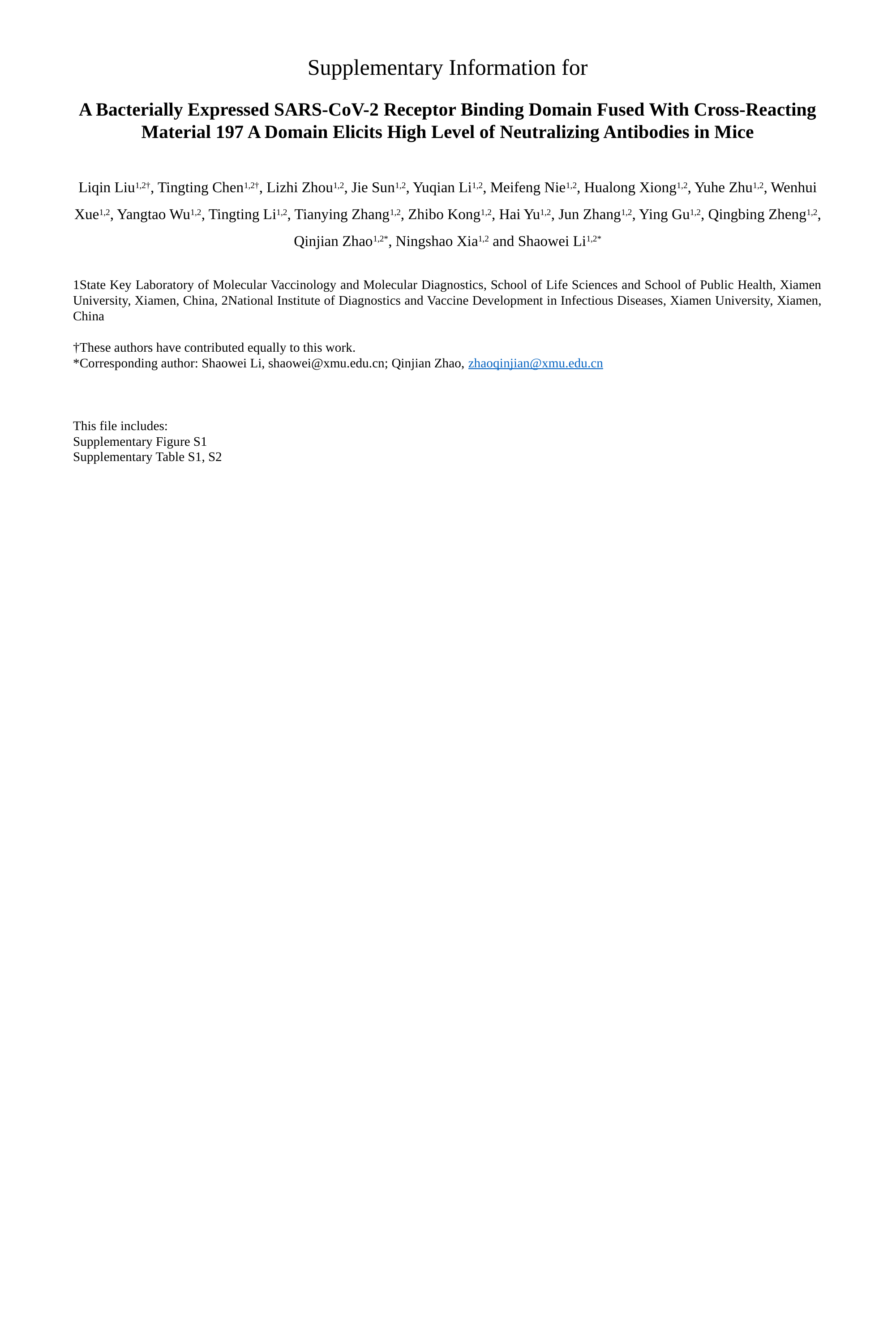

Supplementary Information for
﻿A Bacterially Expressed SARS-CoV-2 Receptor Binding Domain Fused With Cross-Reacting Material 197 A Domain Elicits High Level of Neutralizing Antibodies in Mice
﻿
﻿Liqin Liu1,2†, Tingting Chen1,2†, Lizhi Zhou1,2, Jie Sun1,2, Yuqian Li1,2, Meifeng Nie1,2, Hualong Xiong1,2, Yuhe Zhu1,2, Wenhui Xue1,2, Yangtao Wu1,2, Tingting Li1,2, Tianying Zhang1,2, Zhibo Kong1,2, Hai Yu1,2, Jun Zhang1,2, Ying Gu1,2, Qingbing Zheng1,2, Qinjian Zhao1,2*, Ningshao Xia1,2 and Shaowei Li1,2*
﻿1State Key Laboratory of Molecular Vaccinology and Molecular Diagnostics, School of Life Sciences and School of Public Health, Xiamen University, Xiamen, China, 2National Institute of Diagnostics and Vaccine Development in Infectious Diseases, Xiamen University, Xiamen, China
﻿†These authors have contributed equally to this work.
*Corresponding author: Shaowei Li, shaowei@xmu.edu.cn; Qinjian Zhao, zhaoqinjian@xmu.edu.cn
This file includes:
Supplementary Figure S1
Supplementary Table S1, S2

## Slide 2
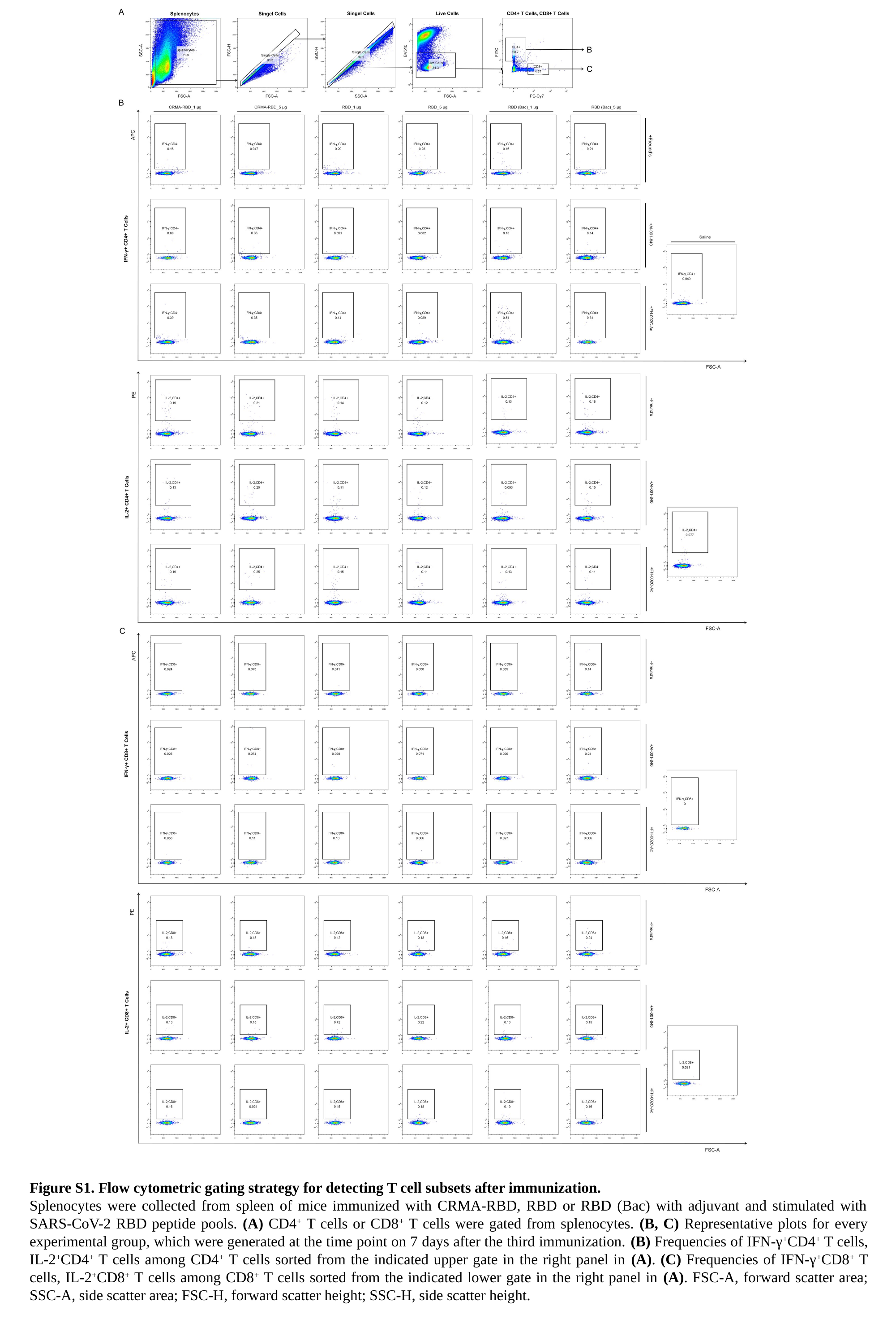

Figure S1. ﻿Flow cytometric gating strategy for detecting T cell subsets after immunization.
Splenocytes were collected from spleen of mice immunized with CRMA-RBD, RBD or RBD (Bac) with adjuvant and stimulated with SARS-CoV-2 RBD peptide pools. (A) CD4+ T cells or CD8+ T cells were gated from splenocytes. (B, C) Representative plots for every experimental group, which were generated at the time point on 7 days after the third immunization. (B) Frequencies of IFN-γ+CD4+ T cells, IL-2+CD4+ T cells among CD4+ T cells sorted from the indicated upper gate in the right panel in (A). (C) Frequencies of IFN-γ+CD8+ T cells, IL-2+CD8+ T cells among CD8+ T cells sorted from the indicated lower gate in the right panel in (A). FSC-A, forward scatter area; SSC-A, side scatter area; FSC-H, forward scatter height; SSC-H, side scatter height.

## Slide 3
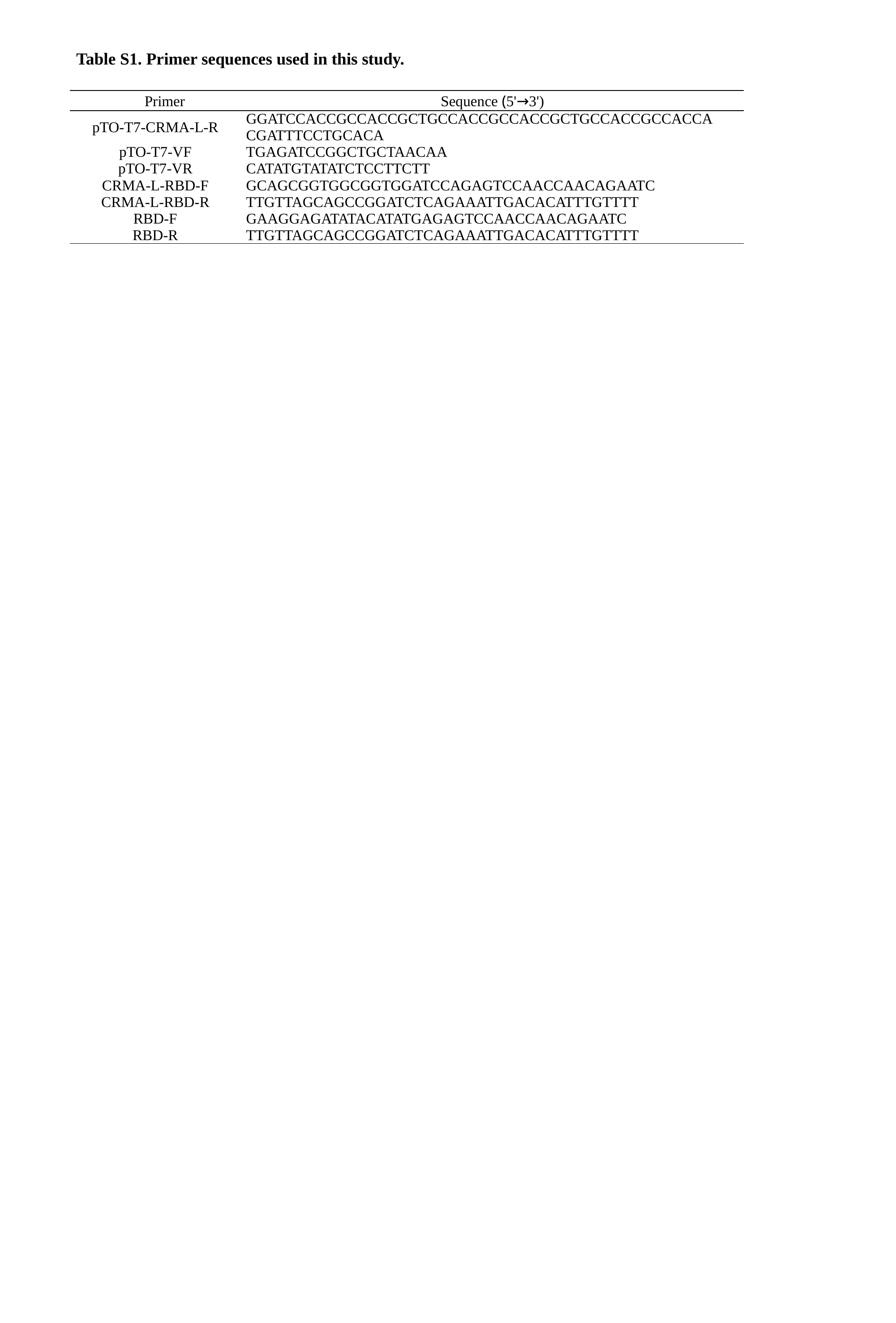

Table S1. Primer sequences used in this study.
| Primer | Sequence (5'→3') |
| --- | --- |
| pTO-T7-CRMA-L-R | GGATCCACCGCCACCGCTGCCACCGCCACCGCTGCCACCGCCACCA CGATTTCCTGCACA |
| pTO-T7-VF | TGAGATCCGGCTGCTAACAA |
| pTO-T7-VR | CATATGTATATCTCCTTCTT |
| CRMA-L-RBD-F | GCAGCGGTGGCGGTGGATCCAGAGTCCAACCAACAGAATC |
| CRMA-L-RBD-R | TTGTTAGCAGCCGGATCTCAGAAATTGACACATTTGTTTT |
| RBD-F | GAAGGAGATATACATATGAGAGTCCAACCAACAGAATC |
| RBD-R | TTGTTAGCAGCCGGATCTCAGAAATTGACACATTTGTTTT |

## Slide 4
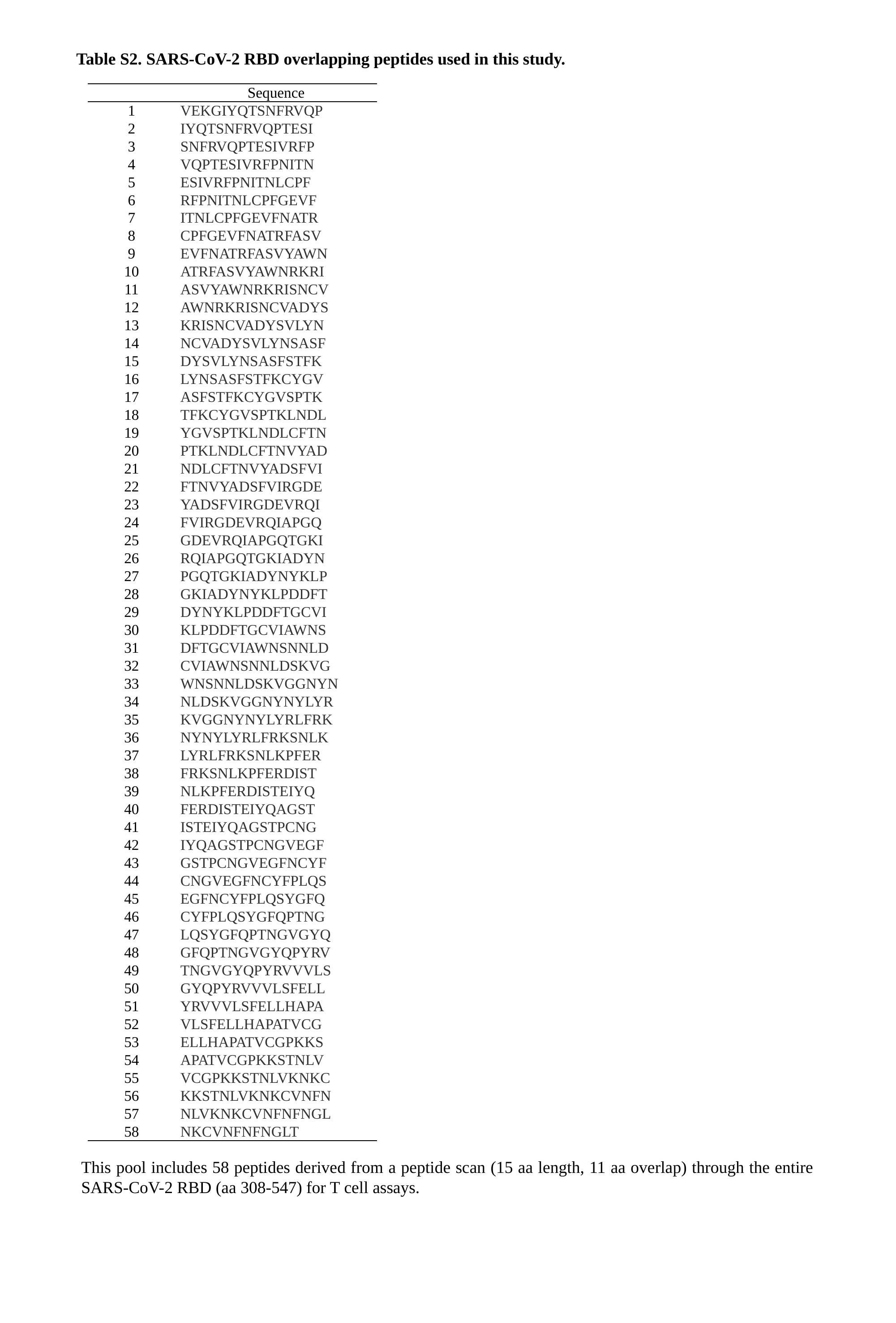

Table S2. SARS-CoV-2 RBD overlapping peptides used in this study.
| | Sequence |
| --- | --- |
| 1 | VEKGIYQTSNFRVQP |
| 2 | IYQTSNFRVQPTESI |
| 3 | SNFRVQPTESIVRFP |
| 4 | VQPTESIVRFPNITN |
| 5 | ESIVRFPNITNLCPF |
| 6 | RFPNITNLCPFGEVF |
| 7 | ITNLCPFGEVFNATR |
| 8 | CPFGEVFNATRFASV |
| 9 | EVFNATRFASVYAWN |
| 10 | ATRFASVYAWNRKRI |
| 11 | ASVYAWNRKRISNCV |
| 12 | AWNRKRISNCVADYS |
| 13 | KRISNCVADYSVLYN |
| 14 | NCVADYSVLYNSASF |
| 15 | DYSVLYNSASFSTFK |
| 16 | LYNSASFSTFKCYGV |
| 17 | ASFSTFKCYGVSPTK |
| 18 | TFKCYGVSPTKLNDL |
| 19 | YGVSPTKLNDLCFTN |
| 20 | PTKLNDLCFTNVYAD |
| 21 | NDLCFTNVYADSFVI |
| 22 | FTNVYADSFVIRGDE |
| 23 | YADSFVIRGDEVRQI |
| 24 | FVIRGDEVRQIAPGQ |
| 25 | GDEVRQIAPGQTGKI |
| 26 | RQIAPGQTGKIADYN |
| 27 | PGQTGKIADYNYKLP |
| 28 | GKIADYNYKLPDDFT |
| 29 | DYNYKLPDDFTGCVI |
| 30 | KLPDDFTGCVIAWNS |
| 31 | DFTGCVIAWNSNNLD |
| 32 | CVIAWNSNNLDSKVG |
| 33 | WNSNNLDSKVGGNYN |
| 34 | NLDSKVGGNYNYLYR |
| 35 | KVGGNYNYLYRLFRK |
| 36 | NYNYLYRLFRKSNLK |
| 37 | LYRLFRKSNLKPFER |
| 38 | FRKSNLKPFERDIST |
| 39 | NLKPFERDISTEIYQ |
| 40 | FERDISTEIYQAGST |
| 41 | ISTEIYQAGSTPCNG |
| 42 | IYQAGSTPCNGVEGF |
| 43 | GSTPCNGVEGFNCYF |
| 44 | CNGVEGFNCYFPLQS |
| 45 | EGFNCYFPLQSYGFQ |
| 46 | CYFPLQSYGFQPTNG |
| 47 | LQSYGFQPTNGVGYQ |
| 48 | GFQPTNGVGYQPYRV |
| 49 | TNGVGYQPYRVVVLS |
| 50 | GYQPYRVVVLSFELL |
| 51 | YRVVVLSFELLHAPA |
| 52 | VLSFELLHAPATVCG |
| 53 | ELLHAPATVCGPKKS |
| 54 | APATVCGPKKSTNLV |
| 55 | VCGPKKSTNLVKNKC |
| 56 | KKSTNLVKNKCVNFN |
| 57 | NLVKNKCVNFNFNGL |
| 58 | NKCVNFNFNGLT |
This pool includes 58 peptides derived from a peptide scan (15 aa length, 11 aa overlap) through the entire SARS-CoV-2 RBD (aa 308-547) for T cell assays.
